# Clinical guidelines and pathways for the management of non-traumatic wrist disorders: A review and synthesis

**DOI:** 10.1177/17589983261422467

**Published:** 2026-02-19

**Authors:** Thomas Mitchell, Joe Palmer, Shaharsha Borkar, Garishma Gajwani, Sandra Tomy, Nimisha Rachel, Nick Hamilton, Benjamin Dean, Sionnadh McLean

**Affiliations:** 1 Centre for Applied Health & Social Care Research (CARe), Sheffield Hallam University, Sheffield, UK; 2 Sheffield Hallam University, Centre for Sports Engineering Research, Sheffield Hallam University, Sheffield, UK; 3 Botnar Research Centre of Musculoskeletal Diseases, 6396 University of Oxford, Oxford, UK; 4 Faculty of Health, School of Allied Health Sciences, Charles Darwin University, Northern Territory, Darwin, Australia

**Keywords:** non-traumatic, wrist injury, wrist pain, scoping review, quality, clinical practice, conservative management

## Abstract

**Introduction:**

Non-traumatic wrist disorders (NTWD) are commonly encountered across a range of healthcare settings. Uncertainty exists regarding optimal management and how this is reflected in care provision through clinical guidelines and pathways. We aimed to identify existing UK-based clinical guidelines and pathways and examine their quality.

**Methods:**

For this review, we searched MEDLINE, PubMed, Embase, CINAHL, ProQuest, Scopus, Web of Science, Google, The National Grey Literature Collection, TRIP, and the National Institute for Health and Care Excellence and undertook targeted outreach and engagement activities with healthcare professional networks. We included clinical practice guidelines and pathways for NTWD published after 2010. The AGREE II tool was used to assess guideline quality based on the domains of topic selection, best practice identification, data collection, and rigor of analysis.

**Results:**

Of 7017 records identified, 15 eligible clinical guidelines and pathways for NTWD management were eligible and included. De Quervain’s tenosynovitis and ganglion cyst were most frequently covered while other NTWD had few or no guidelines or pathways. Variation in the composition and nomenclature of sources was found. Quality assessment using the AGREE II tool identified variability and overall unsatisfactory quality.

**Conclusion:**

There is a lack of high-quality clinical guidelines and pathways for NTWD within the UK healthcare system indicating an obstacle to improvements in healthcare delivery.

## Introduction

Clinical Pathways (CPW) are structured multidisciplinary management plans detailing the sequence of actions in clinical episode using care plans, treatment algorithms, flowcharts and protocols.^1^ They are used to implement broader Clinical Practice Guidelines (CPG) which are grounded in systematic reviews of the evidence.^2^ They improve care quality by facilitating the translation of research into practice, enhancing cost-effectiveness through optimising resource, and standardising care.^3^ An absence of well-defined guidelines is associated with premature and incorrect referrals to hospital-based secondary care, impacting wait times.^4–6^ National Centre for Health and Care Excellence (NICE) guidelines are considered the gold standard due to their rigorous development process and promotion of patient-centred care through evidence-based recommendations. However many MSK CPG have been found to be of poor quality.^7^

Advancements in the management of non-traumatic musculoskeletal (MSK) conditions have lagged behind those of other medical disciplines, leading to an increasing financial and societal burden that is of significant concern to policymakers and governments alike.^8–10^ This has been attributed to the historical reliance on biomedical reasoning, which has fuelled overmedicalisation, resulting in overdiagnosis and the diversion of limited resources toward low-value treatments.^11–13^ Conversely, qualitative research exploring the lived experience of people with MSK spinal and shoulder pain have shaped holistic management strategies, care pathways, and subsequent CPG for these populations.^14–17^ CPG for non-specific low back pain now endorse a biopsychosocial model of care. This approach recommends de-medicalisation by prioritising self-management and non-pharmacological treatments as first-line care whilst adjusting information and messaging to help reset public views about pain and disability, and avoiding imaging when it is unlikely to change management.^18,19^ Wrist pain accounts for an annual consultation prevalence rate of 58 in 10,000 patients seen in primary care in the UK,^20^ and although commonly encountered in all UK healthcare settings,^21,22^ few high-quality resources were found to inform best practice and stakeholders expressed uncertainty about the structure and quality of care provided.^23^ To date, no studies have been conducted examining clinical guidelines and pathways for Non-traumatic Wrist Disorders (NTWD).

To the end of understanding how resources to care for people with NTWD are structured, the aim of this study was to record and appraise clinical guidelines and pathways for NTWD in the UK. Specific objectives were:

• To identify CPW and CPG from grey and published literature and from internally held documents at UK-based health service providers.
• To describe and summarise clinical practice guidelines and care pathways.
• To explore their content through quality assessment using the Appraisal of Guidelines for Research and Evaluation (AGREE II) tool (Supplemental section 1).

## Methods

The review and synthesis were performed in two phases. Firstly, we identified and reviewed clinical guidelines and pathways from a broad array of sources including unpublished and grey literature and those held in local institutions, adhering to the Joanna Briggs Institute (JBI) methodology^24^ for scoping reviews and the PRISMA-ScR checklist.^25^ Secondly, we performed quality assessment. The research team was comprised of a PhD student, four MSc students, academics, and subject area specialists. A protocol for the project was registered on the Open Science Framework on 9^th^ May 2024 prior to gathering source materials (https://osf.io/hmw7q).

Internal institutional approval was granted. Any information or guidelines provided by organisations or institutions were handled with strict confidentiality and used solely for the purposes specified in the research proposal. Anonymisation procedures were implemented to ensure the confidentiality and privacy of all participants and organisations involved.

### Sources and data

Clinical guidelines and pathways were gathered using a pre-defined search strategy. Specific search strategies were employed for Medline, PubMed, CINAHL, and Embase databases (from 2010 to 2024) using combinations of MeSH terms with Boolean operators and truncations to refine the search strategy (Supplemental section 2). ProQuest, Scopus, Web of Science, and Google, The National Grey Literature Collection, TRIP, and the National Institute for Health and Care Excellence were utilised to identify grey literature using refined search terms of “chronic” instead of “nontraumatic,” “hand” instead of “wrist,” and “pain” instead of “disorders”. Local and departmental data were obtained by engaging clinicians and leveraging professional networks, with additional data solicited through online forums, social media, and email requests. Confidentiality and secure data handling were prioritised, with anonymised materials appraised by the research team. Reference lists of the identified sources were reviewed to uncover additional CPW and CPG.

### Inclusion criteria

(1) Sources created after the arbitrary cutoff of 2010. Only the most recent sources were included if multiple versions were available.
(2) Clinical guidelines and pathways which addressed the clinical management of NTWD as defined by Mitchell^23^ (Supplemental section 3).
(3) CPW including care plans, treatment algorithms, flowcharts and protocols related to the management of NTWD.
(4) Sources published in English and produced by UK-based institutions and organisations.

### Exclusion criteria

(1) Sources regarding traumatic injury of the wrist including fractures or dislocations, paediatric populations or congenital wrist disorders.
(2) Sources regarding non-MSK conditions and complex regional pain syndrome (CRPS) (Supplemental section 3). Carpal tunnel syndrome was excluded as it has well established diagnostic and management criteria in contrast to other NTWD.^23^
(3) Patient information sheets.
(4) Primary and secondary research articles.
(5) Non-English sources or CPW or CPG from outside the UK.

### Data charting

Sources meeting the inclusion criteria were described and summarised by four investigators (SB, GG, ST, NR) prior to quality assessment (Table 1).

**Table 1.** General CPW and CPG characteristics.

*ID*	CPW title, short title and link	NTWD condition	Year	Source description	Development method	Development professionals	Target audience	Number of references	Management adjunct
1	De Quervain’s guideline (DeQ Anon)	De Quervain’s	2022	Flowchart^a^	Literature review	Hand therapist	Hand therapists	20	Splinting Activity modification Exercise NSAIDs Hot/cold adjuncts Manual therapy Electrotherapy
	Within-trust CPW- anonymised as per study plan								
2	Extensor carpi ulnaris lesion guideline (ECU Anon)	Other tendinopathies	2020	CPW document^a^ Treatment algorithm	Literature review	Hand therapist	Hand therapists	15	Splint Activity modification Exercise Patient education Hot/cold adjuncts Compression Elevation
	Within-trust CPW- anonymised as per study plan								
3	Ganglion cyst: BMJ best practice (Gang BMJ)	Ganglion	2024	Website CPG resource^a^ Treatment algorithm^b^ Patient information sheet^b^	Literature review and expert panel	Surgeons and physicians	Primary and secondary care clinicians plus service users	40	Activity modification Steroid injection Surgical intervention NSAIDs Patient education Electrotherapy
	https://tinyurl.com/5cserwka								
4	Ganglion cyst removal: Greater Manchester Policy Statement (Gang Manc)	Ganglion	2019	CPW document^a^	Not reported	Not reported	Primary and secondary care clinicians	0	Steroid injection Surgical intervention
	https://tinyurl.com/4fxpnvc4								
5	Hand and wrist: NHS Bournemouth and Poole (Ha/Wr Poole)	De Quervain’s Ganglion Osteoarthritis Tenosynovitis	Not reported	CPW document^a^	Not reported	Not reported	Primary and secondary care clinicians	0	Splinting Activity modification Steroid injection Surgical intervention Patient education Taping Positional advice Rest Aspiration
	https://tinyurl.com/23bd2xvd								
6	Guidelines: De Quervain’s disease/syndrome. Shropshire hospital (DeQ Shrop)	De Quervain’s	Not reported	CPW document^a^	Not reported	Hand therapists	Hand therapists	2	Splinting Activity modification Exercise Surgical intervention Taping Hot/cold adjuncts Manual therapy Positional advice Rest
	https://tinyurl.com/ysb37esa								
7	Hand and wrist pain pathway: by Sussex MSK Partnership Central (Ha/Wr Sus)	De Quervain’s Ganglion Wrist pain Ulnar sided wrist pain	2021	CPW document^a^	Consensus group	Surgeons, GP’s radiologists, hand therapists, service manager	Primary and secondary care clinicians	0	Splinting Steroid injection Surgical intervention Patient education Taping
	https://tinyurl.com/47tjdjv3								
8	Hand/Wrist pathway by NHS Borders (Ha/Wr Bord).	Wrist sprain Osteoarthritis De Quervain’s	Not reported	CPW document^a^ Flowchart^b^	Not reported	Not reported	Primary and secondary care clinicians	0	Splinting Steroid injection Surgical intervention NSAIDs Taping
	https://tinyurl.com/mv24x8vd								
9	Hand and wrist pain pathway by Essex (Ha/Wr Ess)	Wrist sprain Osteoarthritis Tendinopathies Ganglion De Quervain’s	Not reported	CPW document^a^ Flowchart^b^	Not reported	Not reported	Primary and secondary care clinicians	0	Splinting Exercise NSAIDs Hot/cold adjuncts Compression Oral analgesia
	https://tinyurl.com/2c9zw78t								
10	Osteoarthritis in over16s: National Institute for health and care Excellence (OA NICE)	Osteoarthritis	2022	CPG website^a^ Infographic^b^ Patient information sheet^b^	Stakeholder review	Surgeons, GP’s radiologists, academics, service users, special interest groups	Primary and secondary care clinicians	143	Activity modification Exercise Steroid injection NSAIDs Patient education Manual therapy Weight management
	https://tinyurl.com/k3bn7w5y								
11	Tenosynovitis of hand and wrist: BMJ best practice (Teno BMJ)	De Quervain’s Other tendinopathies	2024	Website CPG resource^a^ Treatment algorithm^b^ Patient information sheet^b^	Literature review and expert panel	Surgeons and physicians	Primary and secondary care clinicians plus service users	80	Splinting Activity modification Steroid injection Surgical intervention NSAIDs Rest
	https://tinyurl.com/5n99992w								
12	TIMS GP wrist and hand guide detailed management guidance (Ha/Wr GP)	Osteoarthritis Ganglion De Quervain’s	2021	CPW document^a^	Expert panel	General practitioners	Primary care clinicians	0	Splinting Activity modification Exercise Steroid injection NSAIDs Patient education Positional advice
	https://tinyurl.com/46ryntev								
13	Volar midcarpal instability (VMid Anon)	Midcarpal instability	Not reported	CPW document^a^	Not reported	Hand therapists	Hand therapists	0	Activity modification Exercise
	Within-trust CPW- anonymised as per study plan								
14	Scapholunate instability (SL Anon)	Midcarpal instability	Not reported	CPW document^a^	Not reported	Hand therapists	Hand therapists	0	Activity modification Exercise
	Within-trust CPW- anonymised as per study plan								
15	Distal radio ulnar joint Tear (DRU Anon)	Midcarpal instability	Not reported	CPW document^a^	Not reported	Hand therapists	Hand therapists	0	Splinting Exercise
	Within-trust CPW- anonymised as per study plan								

^a^Primary source description.

^b^Additional source components.

NSAIDs = non-steroidal anti-inflammatory medication.

### Quality assessment

To assess the quality of clinical guidelines and pathways for the management of non-traumatic wrist disorders (NTWD), the AGREE II instrument^26^ was used. Consisting of 23 items assessing sources over six domains (scope and purpose, stakeholder involvement, rigor of development, clarity of presentation, applicability, and editorial independence), the AGREE II provides structured processes to gather objective information about each source (Supplemental section 1) and benefits from an internal consistency between 0.64 and 0.89 with satisfactory inter-rater reliability.^26^ It has been recommended for appraising both clinical guidelines and pathways.^2,27,28^ Four investigators (SB, GG, ST, NR) completed the free online training module available on the AGREE website (www. agreetrust.org). Eligible sources were evaluated independently by the four investigators for methodological quality using the AGREE II tool in an online format hosted on the My AGREE PLUS platform (https://www.agreetrust.org/resource-centre/agree-plus/). Variation in scoring were addressed thorough collaborative discussion to ensure comprehensive coverage of guideline aspects leading to an assignation of an agreed overall assessment score with disagreements being adjudicated by TM.^29^ A ‘scaled domain score’ was determined as a % using the following formula as recommended by Brouwers et al. ^26^:

$$\text{scaled}\;\text{domain}\;\text{score}=\frac{\left(obtained\;score-minimum\;possible\;score\right)}{\left(maximum\;possible\;score-minimum\;possible\;score\right)}\times 100\%$$

In line with recommendations from the AGREE II manual and recent literature, a threshold of 60% was used to assess the quality of the guideline.^3^ Sources were rated as ‘high quality’ when a 60% threshold was exceeded in 5 or more domains, ‘average quality’ when 3 or 4 domains scored more than 60%, and ‘low quality’ where less than 2 domains scored greater than 60%. Overall mean quality scores for each domain and guideline were reported.

## Results

**Figure 1.**
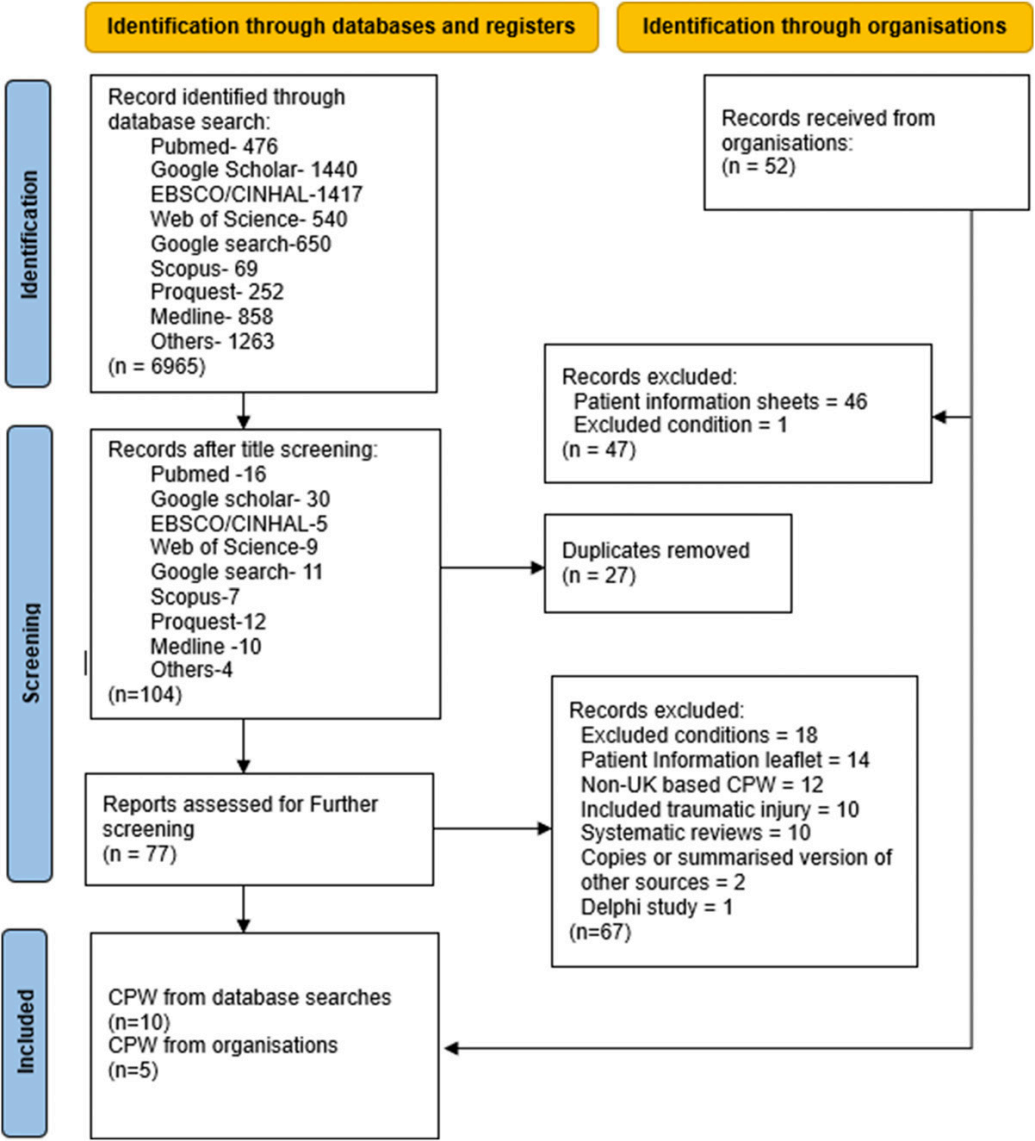
PRISMA diagram detailing the identification of sources through databases and local institutions.

The primary search strategy and appeal for local CPW and CPG identified 7017 sources (Figure 1). No sources were derived from Google Scholar, ProQuest, Scopus, or national grey literature collections. Title screening and removal of duplicated items returned 77 sources for full-text evaluation. Organisational email handouts contributed 52 records. After matching sources to exclusion criteria 10 clinical guidelines and pathways for NTWD were returned from databases and 5 CPW received from local organisations, totalling 15 records for analysis.

Table 1 provides information on general characteristics for each CPW and CPG. Grouped data for the sources can be found in Supplemental section 4 and will be referred to using square brackets and italics. The conditions served by the greatest number of sources were De Quervain’s Tenosynovitis (n = 8; [*1,5-9,11-12*]) and ganglion (n = 8; [*3-5,7,9,12*]), other tendinopathies (n = 4; [*2,5,9,11*]), osteoarthritis (n = 4; [*5,8,9,12*]) and wrist pain/strain (n = 2; [*7,8*]). Single or no sources were found for other non-traumatic wrist conditions. The primary source description for 11 of the records was a CPW document [*2,4-9,12-15*]; three were described as Website CPG resources [*3,10,11*] and one comprised a flowchart [*1*]. The three website CPGs contained additional source components of patient information sheets, and two sources contained flowcharts and algorithms [*3,11*], and one included an infographic [*10*]. Nine of the sources did not contain any references [*4,5,7-9,12-15*], with the remaining 6 sources ranging from 2 to 143 references. No year of publication was found on 7 CPW documents [*5,6,8,9,13-15*], and 8 CPW did not include information on the method of development [*4-6,8,9,13-15*] with the remaining sources developed through literature review [*1,2*], literature review and expert panel [*3,11*] and by consensus panel [*7*]. The most common professional grouping to create CPW were Hand therapists (n = 6; [*1,2,6,13-15*]), though 4 sources did not report their composition [*4,5,8,9*] and the remaining were mixed clinician groups [*3,7,10,11*], and General Practitioners [*12*]: of note, two sources also included service users [*3&11*]. A single CPW was targeted toward primary care settings/clinicians [*12*], with six focussed on secondary care management and six targeting both primary and secondary care. Substantive heterogeneity was recorded in the treatment options recommended for NTWD with 84 adjuncts mentioned across 18 categories. Fifty-one treatments were spread across 6 categories comprised of splinting (n = 10; [*1,2,5-9,11,12,15*]), activity modification (n = 10; [*1-3, 5, 6, 10-14*]), exercise (n = 9; [1,2,6,9,10,12-15]), steroid injection (n = 8; [*3-5,7,8,10-12*]), surgery (n = 7; [*3-8,11*]) and non-steroidal anti-inflammatory medication (n = 7; [*1,3,8-12*]). Specific unique treatments included aspiration, analgesia, oral medications, and weight management [*2,5,9.10*].

Table 2 displays scaled domain scores and overall quality assessment for each source. ‘Osteoarthritis in over 16’s’ by NICE demonstrated a ‘High’ quality rating [*10*] reflecting its comprehensive scope, methodological rigor, and applicability, and two others were rated ‘average’ [*3 & 11*] were both produced by ‘British Medical Journal Best Practice Guides’ (BMJ) demonstrating clarity, focus, and stakeholder engagement. The CPG’s scored better in overall assessment scores compared with CPW. The remaining eleven CPW [*2,4-9,12-15*] and one flowchart [*1*] received ‘Low’ quality ratings with mean overall score between 23.3% and 28% assigned to Wr/Ha GP [*12*], an anonymised CPW covering De Quervain’s, Extensor carpi ulnaris tendinopathy, scapholunate instability, distal radio-ulnar joint instability and volar midcarpal instability [*1,2,13-15*]. This level of quality indicates a lack of methodological rigor, no stakeholder involvement, and poor clarity of presentation. Of the selected guidelines, the overall highest ratings were from established national professional and government funded organisations and the lowest from local institutions.

**Table 2.** Quality assessment of included Clinical pathways and guidelines.

Reference number	Short title	Scope and purpose %	Stakeholder involvement %	Rigor of development %	Clarity of presentation %	Applicability %	Editorial independence %	Mean overall score %	Quality rating
1	DeQ Anon	18.0	13.8	22.9	65.2	20.8	4.1	24.1	Low
2	ECU Anon	18.0	13.8	22.9	65.2	20.8	4.1	24.1	Low
3	Gang BMJ	61.1	58.3	47.9	81.9	33.3	62.5	57.5	Average
4	Gang Manc	73.6	30.5	57.8	56.9	39.5	41.6	50.0	Low
5	Ha/Wr Poole	16.6	8.3	27.6	41.6	19.7	58.3	28.7	Low
6	DeQ Shrop	31.9	16.6	9.3	44.4	11.4	8.3	20.3	Low
7	Ha/Wr Sus	31.9	25	24.4	62.5	28.1	62.5	39.1	Low
8	Ha/Wr Bord	55.5	43.0	43.7	45.8	13.5	56.2	43.0	Low
9	Ha/Wr Ess	58.3	40.2	35.4	63.8	27.0	60.4	47.5	Low
10	OA NICE	91.6	84.7	87.5	95.8	75	91.6	87.7	High
11	Teno BMJ	66.0	72	41.6	80.5	37.5	81.2	63.1	Average
12	Ha/Wr GP	40.2	18	10.9	48.6	13.5	83	35.7	Low
13	VMid Anon	19.4	8.3	11.4	70.8	22.9	35.4	28.0	Low
14	SL Anon	19.4	8.3	11.4	70.8	22.9	35.4	28.0	Low
15	DRU Anon	19.4	8.3	11.4	70.8	22.9	35.4	28.0	Low
	**Mean ± SD**	41.4 ± 24.5	29.9 ± 24.8	31.1 ± 21.9	64.3 ± 15.2	27.3 ± 15.6	43.0 ± 27.9		

DeQ Anon = De Quervain’s: Within-trust CPW; ECU Anon = Extensor Carpi Ulnaris lesion Guideline: Within trust; Gang BMJ = Ganglion Cyst: BMJ Best Practice; Gang Manc = Ganglion Cyst Removal: Greater Manchester Policy Statement; Ha/Wr Poole = Hand and wrist: NHS Bournemouth and Poole; Ha/Wr Sus = Hand and Wrist Pain Pathway: by Sussex MSK Partnership Central; Ha/Wr Bord = Hand/Wrist Pathway by NHS Borders; Ha/Wr Ess = Hand and Wrist Pain Pathway by Essex; Osteoarthritis in over16s: OA NICE = National Institute for Health and Care Excellence; Teno BMJ = Tenosynovitis of hand and wrist: BMJ Best Practice; Ha/Wr GP = TIMS GP wrist and hand guide detailed management guidance; VMid Anon = Volar Midcarpal Instability: within trust CPW; SL Anon = Scapholunate Instability within trust CPW; DRU Anon = Distal Radio Ulnar Joint Tear within trust CPW.

The domain of *Clarity of Presentation* had the highest overall values with 64.3% ± 15.2% which correlated with least variability represented by the standard deviation (SD) and related to the extent that specific and clear recommendations with well-documented management options were present. *Clarity of Presentation* assesses and evaluates the presentation and format of the guidelines. Across the sources, key recommendations were specific and often presented in bulleted form, flowcharts, and easily identifiable diagrams. This is a valuable strength in allowing clinicians from a variety of disciplines to access resource. No other domain reached over 50% in scaled score, and lowest scoring was *Applicability* which assesses the likelihood of the guidelines being applied in practice, demonstrated the next lowest variability in SD (27.3% ± 15.6). Consistently higher SDs were found in the domains *Scope and Purpose* (41.4 ± 24.5), *Stakeholder Involvement* (29.9 ± 24.8), *Rigor of Development* (31.1 ± 21.9) and *Editorial Independence* (43.0 ± 27.9). Most guidelines did not provide evidence of pilot testing with target users, nor did they demonstrate inclusion of user views and preferences. Additionally, the composition of the guideline development group and the roles of its members were frequently omitted. The *Rigor of Development* domain appraisal often indicated a lack of a description of an external review process by experts before publication, and lack of specified procedures for updates. Some components - such as health benefits, side effects, and risks - were addressed, but others were omitted, resulting in a lower score for this domain.

In summary, the guidelines performed best in *Clarity of Presentation* but were particularly weak in *Applicability* and *Rigor of Development*, with significant shortcomings in S*takeholder Involvement* and *Editorial Independence*.

## Discussion

By recording and appraising clinical guidelines and pathways for non-traumatic wrist conditions, our study demonstrated significant variation in the availability, quality, and content of CPGs and CPWs, with NICE and BMJ guidelines demonstrating ‘gold-standard’ examples. There were relatively few resources for non-traumatic wrist conditions in the UK (n = 15), and our searches captured only a further 12 from non-UK origin. De Quervain’s tenosynovitis, ganglion and other tendinopathies and osteoarthritis were best supported while other conditions lacked resource to guide management. Shortcomings in the delivery of high-value care for wrist and hand conditions have been identified by different research groups^30,31^ and this evaluation of CPG and CPW allows insights into the quality of available resource and may be used to frame future development.

The overall dataset reveals issues with the nomenclature of CPW and CPG. Several locally held CPW’s are incorrectly labelled as guidelines [*1,2 & 6*] while patient information sheets were frequently returned from database searches and local departments. The importance of good quality patient information cannot be overstated, but not in lieu of overarching clinical guidelines and pathways. The current study assessed freely available and donated local resources, yet the absence of any CPW or CPG on the websites of the British Association of Hand Therapists and the British Society for Surgery of the Hand is notable,^32,33^ and contrasts with extensive provision for trauma cases, carpal tunnel syndrome and complex regional pain syndrome. Interestingly both groups websites feature patient-facing information about specific NTWD as standalone resources.^34^ The clustering of resource around certain conditions indicates many NTWD would benefit from improved care provision guidance. The case for adopting recommended structures for CPG and CPW to drive quality improvement^1^ is supported in part by the variety in the composition of sources. A myriad of treatment options ranging from surgical, pharmacologic and injections to conservative rehabilitation interventions and passive adjuncts may be used to treat NTWD. This is consistent with earlier studies into the management of these conditions and reflects uncertainty in best practice.^23,35^ Major omissions in the sources included the absence of an expected timeline to guide navigation through care pathways and a lack of recommended outcome measures to assess patients’ functional status.

It was expected that AGREE II scores for the CPG performed better than CPW due to the roles NICE and the BMJ have in shaping care. Being created by a national body did not necessarily guarantee better standards as the GP-lead TIMS group ‘Wrist and Hand resource’ had the lowest overall quality score (23.3%). There was considerable variation in mean overall *Scope and Purpose* scores suggesting that while some clearly define their objectives and the health questions they address, others lack specificity and comprehensive coverage. *Stakeholder involvement* was another area of notable disparity, with CPGs showing robust engagement at best, while CPWs often lacked the involvement of professional groups and service users in their development. The *Rigour of Development* parameter revealed notable differences, with some guidelines employing thorough, systematic methodologies and robust evidence collection, while others lacked such rigour, undermining their credibility and reliability. *Clarity of presentation* was generally high across most guidelines suggesting clear and accessible information. Lower scores in *Applicability* were observed across all groups mirroring challenges many guidelines face in terms of practical applicability, including considerations of facilitators and barriers to implementation in clinical settings. Lastly, *Editorial independence*—which reflects the extent to which guidelines are free from funding body influence and clearly declare conflicts of interest—showed considerable heterogeneity, with scores ranging from 4.1 to 91.6, indicating a frequent lack of transparency.

To align the management of individuals with wrist conditions with the strategic objectives of the NHS Long Term Plan, specifically in delivering person-centred and value-based healthcare,^36^ key developments include a priority-setting initiative^37^ and the establishment of a core outcome set.^31^ These should be integrated within a biopsychosocial model of care for individuals with NTWD. Future qualitative studies are indicated to effectively inform the development of new CPW and CPG.

## Strengths and limitations

The study benefited from an *a priori* structure, achieved through pre-registration of the protocol on the Open Science Framework, and adhered to the JBI criteria, PRISMA scoping review guidelines, and the use of the validated AGREE II tool for quality assessment. The use of four appraisers to independently review all CPW mitigated the risk of bias. Direct appeal to local institutions for non-public sources was successful**.** A limitation was that only sources written in English were included.

## Conclusion

There is a lack of high-quality clinical pathways and guidelines for non-traumatic wrist disorders (NTWD) within the UK healthcare system, presenting an obstacle to improvements in care delivery. To ensure consistent and effective management, more robust, evidence-based guidelines are needed to optimise healthcare delivery and improve patient outcomes for NTWD.

## Supplemental material

Supplemental material - Clinical guidelines and pathways for the management of non-traumatic wrist disorders: A review and synthesis Supplemental material for Clinical guidelines and pathways for the management of non-traumatic wrist disorders: A review and synthesis by Thomas Mitchell, Joe Palmer, Shaharsha Borkar, Garishma Gajwani, Sandra Tomy, Nimisha Rachel, Nick Hamilton, Benjamin Dean, Sionnadh McLean in Hand Therapy
