# Supplementary material for: Clinical guidelines and pathways for the management of non-traumatic wrist disorders: A review and synthesis
Source: Hand Ther. 2026 Feb 19:17589983261422467. Online ahead of print. doi: 10.1177/17589983261422467 (PMC12920155; doi:10.1177/17589983261422467)
Supplement: Supplemental material - Clinical guidelines and pathways for the management of non-traumatic wrist disorders: A review and synthesis [file sj-pdf-1-hth-10.1177_17589983261422467.pdf]

Supplementary section 1

Scope and Purpose	
1	The overall objective(s) of the guideline is (are) specifically described.
2	The health question(s) covered by the guideline is (are) specifically described.
3	The population (patients, public, etc.) to whom the guideline is meant to apply is specifically described.
Stakeholder involvement	
4	The guideline development group includes individuals from all relevant professional groups.
5	The views and preferences of the target population (patients, public, etc.) have been sought.
6	The target users of the guideline are clearly defined.
Rigor of development	
7	Systematic methods were used to search for evidence.
8	The criteria for selecting the evidence are clearly described.
9	The strengths and limitations of the body of evidence are clearly described.
10	The methods for formulating the recommendations are clearly described.
11	The health benefits, side effects, and risks have been considered in formulating the recommendations.
12	There is an explicit link between the recommendations and the supporting evidence.
13	The guideline has been externally reviewed by experts prior to its publication.
14	A procedure for updating the guideline is provided.
Clarity of presentation	
15	The recommendations are specific and unambiguous.
16	The different options for management of the condition or health issue are clearly presented.
17	Key recommendations are easily identifiable.
Applicability	
18	The guideline describes facilitators and barriers to its application.
19	The guideline provides advice and/or tools on how the recommendations can be put into practice.
20	The potential resource implications of applying the recommendations have been considered.
21	The guideline presents monitoring and/or auditing criteria.
Editorial independence	
22	The views of the funding body have not influenced the content of the guideline.
23	Competing interests of guideline development group members have been recorded and addressed.

AGREE II = Appraisal of Guidelines for Research and Evaluation.

Supplementary section 2: Search terms 08.06.2024

Databases	Keywords
**Pubmed/MEDLINE**<br><br>**ProQuest**	(*Overuse OR chronic OR Nonspecific) AND (Wrist OR Hand OR Carpal OR Radioulnar OR Ulnocarpal) AND ("Management") OR treatment OR Rehabilitation) NOT<br>(Surgery OR Operation Or surgical procedure)<br>("wrist") OR wrist pain OR Wrist disorders) AND management and rehabilitation and physical therapy) NOT (surgery OR fractures OR Traumatic)
**EBSCO/CINAHL**	Tenosynovitis AND (guidelines or protocols or practice guideline or clinical practice guideline) OR BMJ best practice<br>NOT (management or intervention or therapy) NOT (Surgery OR operation or surgical procedure or surgical treatment or operative

Supplementary section 3

Included conditions	Excluded conditions
Ganglion	Base of thumb and hand osteoarthritis
Ulna sided wrist pain	Carpal fractures
Dequervain's Intersection and other tendinopathies	Carpal tunnel syndrome
Carpal osteoarthritis	Sclerosis
Carpal instabilities	Rheumatoid arthritis and other inflammatory arthritides
Other entrapment neuropathies	Chronic regional pain syndrome or neuropathic disorders
Unknown	

Supplementary section 4: Grouped CPW and CPG characteristics

	Characteristic	*N*	Reference ID
**NTWD condition**	De Quervain's	8	*1, 5-9, 11-12*
	Ganglion	8	*3-5, 7, 9, 12*
	Other tendinopathies	4	*2, 5, 9 11*
	Osteoarthritis	4	*5, 8, 9, 12*
	Ulnar-sided wrist pain	2	*7, 8*
	Wrist pain/strain	1	*7*
	Distil radio ulna joint	1	*15*
	Volar midcarpal instability	1	*13*
	Scapholunate instability	1	*4*
**Source components**	CPW document	11	*2, 4-9, 12-15*
	Website CPG resource	3	*3, 10, 11*
	Flowchart	3	*1, 8, 9*
	Includes patient information sheet	3	*3, 10, 11*
	Treatment algorithm	1	*11*
**Development method**	Not reported	8	*4-6, 8, 9, 13-15*
	Literature review	2	*1, 2*
	Literature review and expert panel	2	*3, 11*
	Expert panel	1	*12*
	Consensus group	1	*7*
	Stakeholder review	1	*10*
**Development group composition**	Hand therapists	6	*1, 2, 6, 13-15*
	Not reported	4	*4, 5, 8, 9*
	Surgeons and physicians	2	*3, 11*
	Mixed professional groups	2	*7, 10*
	General practitioners	1	*12*
**Target user/setting**	Primary and secondary care clinicians	6	*6-7, 9-12*
	Secondary care hand therapists	6	*1, 2, 6, 13-15*
	Primary care clinicians	1	*12*
**Recommended interventions**	Splinting	10	*1, 2, 5-9, 11, 12, 15*
	Activity modification	10	*1-3, 5, 6, 10-14*
	Exercise	9	*1, 2, 6, 9,10, 12-15*
	Steroid injection	8	*3-5, 7, 8, 10-12*
	Surgical intervention	7	*3-8, 11*
	Non-steroidal anti-inflammatory medication	7	*1, 3, 8-12*
	Patient education	6	*1, 2, 5, 7, 10, 12*
	Taping	4	*5-8*
	Hot/cold adjuncts	4	*1, 2, 6, 9*
	Manual therapy	3	*1, 6, 10*
	Positional advice	3	*5, 6, 12*
	Electrotherapy (various)	2	*1, 2*
	Rest	3	*5, 6, 11*
	Compression	2	*2, 9*
	Oral analgesia	1	*9*
	Elevation	1	*2*

Weight management	1	*10*
Aspiration	1	*5*
